# A Novel Uncertainty Management Approach for Air Combat Situation Assessment Based on Improved Belief Entropy

**DOI:** 10.3390/e21050495

**Published:** 2019-05-14

**Authors:** Ying Zhou, Yongchuan Tang, Xiaozhe Zhao

**Affiliations:** School of Electronics and Information, Northwestern Polytechnical University, Xi’an 710072, China

**Keywords:** Dempster–Shafer evidence theory (DST), air combat situation assessment, belief entropy, uncertainty management, uncertainty measure

## Abstract

Uncertain information exists in each procedure of an air combat situation assessment. To address this issue, this paper proposes an improved method to address the uncertain information fusion of air combat situation assessment in the Dempster–Shafer evidence theory (DST) framework. A better fusion result regarding the prediction of military intention can be helpful for decision-making in an air combat situation. To obtain a more accurate fusion result of situation assessment, an improved belief entropy (IBE) is applied to preprocess the uncertainty of situation assessment information. Data fusion of assessment information after preprocessing will be based on the classical Dempster’s rule of combination. The illustrative example result validates the rationality and the effectiveness of the proposed method.

## 1. Introduction

Artificial intelligence technology has brought new weapons to intelligent air combat, e.g., the ALPHA software system [1,2,3]. In air combat situation assessment, the decision-making procedure of pilots and unmanned combat aerial vehicle is deeply dependent on intelligent information processing methods [4,5]. Air combat situation assessment often includes threat assessment such as the prediction of enemy’s military intentions. Existing researches mainly include target threat assessment between the two sides basing on the space situation. In [6], the naive Bayes method is used to design the air combat situation assessment method. In [7,8], the Bayesian networks are used to model the situation assessment environment to obtain a better understanding of the battlefield scenario. A fuzzy logic-based situation assessment system is developed to help the pilot make the right decision in complex air scenarios where there may be multiple friendly aircrafts and/or enemy aircrafts [9]. In [10], the situation assessment knowledge of fighter pilots in air combat is applied to model a human situation assessment model in Bayesian networks, which is a new perspective to improve the performance of fighter pilots in air combat.

The aforementioned methods can inherit the advantages of the corresponding intelligent algorithm. However, more attention should be addressed to the data fusion process of the heterogeneous information coming from multiple sources [11]. Due to the dynamic complexity and high real-time characteristic of the battlefield environment, decision-making basing on only the information of a single sensor at a single time cannot accurately depict the complex situation of air combat. The application of information fusion technology can contribute to a better understanding of a situation in a battlefield and provide technical support for real-time decision-making for the pilots and commanders. This paper studies the uncertain information fusion of air combat situation assessment under the framework of evidence theory [12,13] since DST is a typical method for uncertain information processing [14,15,16,17,18].

To address the uncertainty in air combat situation assessment, an improved belief entropy (IBE)-based situation assessment method in the evidence theory framework is proposed. Firstly, evidence modeling will be based on an understanding of air combat situations according to the knowledge in the military domain. Secondly, the uncertainty of each evidence is measured and applied to evidence modification. The uncertainty measure for evidence is based on the IBE [19], which is another hot topic in the DST framework [20,21,22,23,24]. After that, Dempster’s rule of combination is chosen for information fusion after evidence modification. Finally, real-time decision-making in air combat situation assessment aided by intelligent information fusion will be used to support the pilots and commanders. The illustrative example shows the effectiveness and availability of the IBE-based air combat situation assessment method.

This rest of this paper is organized as follows. The preliminaries are introduced in Section 2. In Section 3, an IBE-based situation assessment method for air combat is proposed. A numerical example of applying the proposed method for real-time decision-making in air battlefield environment is presented in Section 4. Some open issues of the current work are discussed in Section 5. Section 6 presents the conclusion and possible future work.

## 2. Preliminaries

### 2.1. Dempster–Shafer Evidence Theory

Some basic definitions in DST are presented as follows [12,13].

**Definition** **1.** *Assume that $\Omega =\left\{\theta_{1},\theta_{2},\ldots ,\theta_{i},\ldots ,\theta_{N}\right\}$ is a nonempty set with N mutually exclusive and exhaustive events, *Ω* is the frame of discernment (FOD). The power set of *Ω* consists of $2^{N}$ elements denoted as follows:*

(1) $$2^{\Omega }=\left\{\begin{array}{c} \emptyset ,\left\{\theta_{1}\right\},\left\{\theta_{2}\right\},\ldots ,\left\{\theta_{N}\right\},\left\{\theta_{1},\theta_{2}\right\},\\ \ldots ,\left\{\theta_{1},\theta_{2},\ldots ,\theta_{i}\right\},\ldots ,\Omega \end{array}\right\}.$$

**Definition** **2.** *A mass function m is a mapping from the power set $2^{\Omega }$ to the interval [0,1]. m satisfies*

(2) $$m\left(\emptyset \right)=0,\sum_{A\in \Omega }m\left(A\right)=1.$$

*If $m\left(A\right)>0$, then A is called a focal element. $m\left(A\right)$ indicates the support degree of the evidence on the proposition A.*

**Definition** **3.** *In DST, two independent mass functions $m_{1}$ and $m_{2}$ can be fused with Dempster’s rule of combination:*

(3) $$m\left(A\right)=\left(m_{1}⊕m_{2}\right)\left(A\right)\;=\frac{1}{1-k}\sum_{B\cap C=A}m_{1}\left(B\right)m_{2}\left(C\right)$$

*where k is a normalization factor defined as follows:*

$$k=\sum_{B\cap C=\emptyset }m_{1}\left(B\right)m_{2}\left(C\right).$$

### 2.2. Improved Belief Entropy

Belief entropy in the DST framework is still an open issue [25,26,27]. Based on Deng entropy [28] and the weighted belief entropy [29], an IBE is proposed in [19], shown as follows:

(4) $$E_{ibe}\left(m\right)=-\sum_{A\subseteq \Omega }m\left(A\right)\log_{2}\left(\frac{m\left(A\right)}{2^{\left|A\right|}-1}e^{\frac{\left|A\right|-1}{\left|\Omega \right|}}\right)$$

where Ω is the FOD, $\left|A\right|$ denotes the cardinality of the focal element *A*, and $\left|\Omega \right|$ is the number of elements in the FOD.

## 3. Improved Belief Entropy-Based Situation Assessment Method for Air Combat

**Figure 1:**
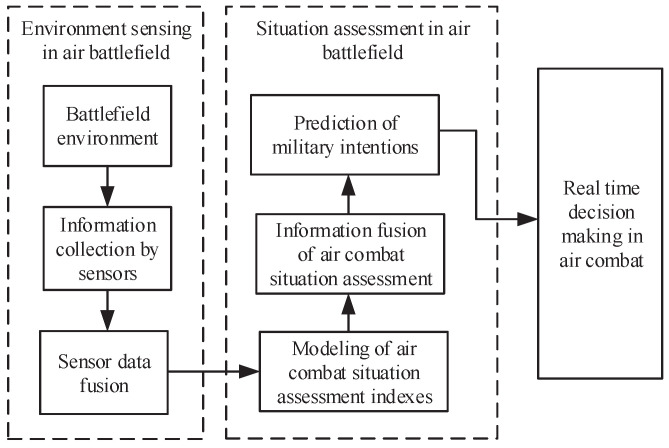
A typical framework of air combat situation assessment.

A typical framework of situation assessment in air combat is presented in Figure 1 [30,31], where there are mainly three main processes: environment sensing in air battlefields, situation assessment of air battlefields, and real-time decision-making.

**Figure 2:**
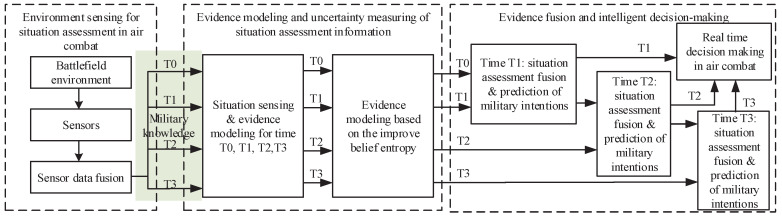
The proposed framework of air combat situation assessment based on the improved belief entropy (IBE).

The framework of the IBE-based situation assessment method for air combat is proposed in Figure 2. Three main processes (including seven steps)—environmental awareness for air combat situation assessment, evidence modeling and uncertainty measures for situation assessment, and air combat situation assessment and fusion-based intelligent air combat decision-making—are designed in the proposed method.

The seven steps in the IBE-based situation assessment method for air combat are described as follows.

- Step 1: battlefield environment information modeling
  All the information about the two sides in the battlefield, the weather, the terrain, the international political situation, and the military expert assessment information should be included in the model of the battlefield environment.
- Step 2: sensor information acquisition
  Battlefield information acquisition will be based on many kinds of sensors, including satellites, air reconnaissance aircraft, ground-based radar, airborne radars, and intelligence agent.
- Step 3: sensor data fusion
  The heterogeneous sensor information acquired from the previous step will be fused for air combat situation assessment. The sensor data fusion method needs to be chosen cautiously, which is another important issue.
- Step 4: air combat situation understanding and evidence modeling
  Sensor data fusion results shall be modeled in the DST framework based on air combat situation understanding. The knowledge in the military domain includes clustering analysis of multi-enemy targets, behavior analysis of the enemy, etc.
- Step 5: IBE-based uncertainty measure and preprocessing of the evidence for situation assessment
  Before applying evidence fusion of the situation assessment information, the uncertainty of each evidence is measured and applied for evidence modification. If evidence has a high degree of uncertainty, which is represented by a high value of IBE, then the reliability of the evidence is low, which means the evidence makes a small contribution to the final decision. Based on this cautious rule, the preprocessing of the evidence based on the uncertainty measure results will be based on the following function:
  (5) $$m_{w}\left(\cdot \right)=\sum_{i=0}^{n}m_{i}\left(\cdot \right)\frac{e^{-E_{ibe}\left(m_{i}\right)}}{\sum_{i=0}^{3}e^{-E_{ibe}\left(m_{i}\right)}}$$
  where $m_{w}\left(\cdot \right)$ means the modified evidence after preprocessing, $m_{i}$ (i=1,2,…,n) is the *i*th piece of evidence coming from air combat situation understanding and evidence modeling, and $E_{ibe}$ is the IBE defined in Equation (4).
- Step 6: information fusion of air combat situation assessment and the prediction of military intention
  In this paper, Dempster’s rule of combination is chosen for information fusion after evidence modification. The prediction of military intention will be based on the fused situation assessment information. If the evidence modification is based on (n+1) (n=1,2,3…) pieces of evidence, the time of information fusion for the modified evidence is *n*.
  (6) $$m{\left(\cdot \right)}_{\left(0,1,2,\ldots ,n\right)}={\left({\left({\left(m_{w}⊕m_{w}\right)}_{1}⊕m_{w}\right)}_{2}⊕\ldots \right)}_{n}\left(\cdot \right)$$
  where ⊕ refers to the information fusion of the modified evidence $m_{w}\left(\cdot \right)$, which is based on Dempster’s rule of combination in Equation (3).
- Step 7: real-time decision-making of air combat
  Real-time decision-making in air combat situation assessment aided by the aforementioned intelligent information fusion method will be used to support the pilots and commanders.

## 4. Example

### 4.1. Problem Description

**Figure 3:**
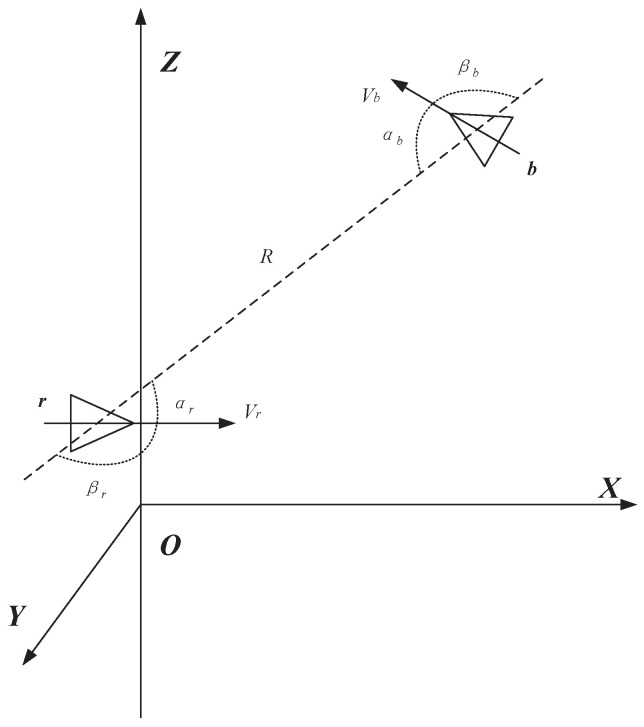
The sketch map of two fighter planes in the battlefield environment.

In air combat, assume that there are two fighter planes in the battlefield environment, as shown in Figure 3. In the space OXYZ, two fighter planes are denoted as *r* and *b*, the distance is *R*, the azimuth angle is α, the target entry angle is β, and *V* represents the speed.

The military intention of the opposite fighter plane can be assessed and modeled in the DST framework.

Assume that the threshold of the belief for the intelligence-aided decision support system in the air combat situation assessment is 90%. In other words, if the final decision of a pilot is based on an intelligent method provided by the decision support system, then the belief of the proposal for the intelligent decision-making should be no less than 90%. The high belief condition set for the intelligent decision support system is to ensure high reliability in possible practical engineering applications. This is reasonable because it is a matter of life and death. If the intelligent aided decision support system has not reached its threshold for intelligent decision-making, then the belief on each candidate should be presented to human beings for reference.

### 4.2. Implementation Steps

Steps 1–3. Battlefield environment information modeling, sensor information acquisition in battlefields, and the corresponding sensor data fusion are key issues corresponding to environment sensing of situation assessments in air combat. Related work can be found in [32,33,34]. This paper will focus on an accurate fusing method for the evidence of the air combat situation assessment. Thus, the illustrative example will focus on Steps 4–7.

Step 4: air combat situation understanding and evidence modeling Assume that there are four types of battlefield situation: the attack situation, denoted as SA1, the defence situation, denoted as SA2, the escape situation, denoted as SA3, and the feint situation, denoted as SA4. Thus, the FOD for the types of battlefield situation is $\Omega =\left\{SA1,SA2,SA3,SA4\right\}$. The evidence modeling after environment sensing for Step 4 is shown as follows.

- At time T0, the sensor reports event $E_{0}$, and a fighter plane appears. The distance is 100 km, the speed is Mach 1.2, and the military intentions are not clear. The evidence modeling based on the military knowledge is $m_{0}=\left\{SA1,SA2,SA3,SA4\right\}=\left\{0.25,0.25,0.25,0.25\right\}$.
- At time T1, the sensor reports event $E_{1}$, and the fighter plane is approaching at Mach 1.8. The evidence modeling based on military knowledge is $m_{0}=\left\{SA1,SA2,SA3,SA4\right\}=\left\{0.3,0.2,0.2,0.3\right\}$.
- At time T2, the sensor reports event $E_{2}$, and the fighter plane is approaching at Mach 2.2. The evidence modeling based on the military knowledge is $m_{0}=\left\{SA1,SA2,SA3,SA4\right\}=\left\{0.4,0.1,0.2,0.3\right\}$.
- At time T3, the sensor reports event $E_{3}$, and the fighter plane is still approaching at a high speed. In addition, its fire control radar is turned on, the attack situation becomes obvious, and the evidence modeling based on the military knowledge is $m_{0}=\left\{SA1,SA2,SA3,SA4\right\}=\left\{0.7,0.1,0.1,0.1\right\}$.

The mass functions of the aforementioned situation assessment information in air combat are shown in Table 1.

Step 5: IBE-based uncertainty measure and preprocessing of the evidence for situation assessment The uncertain degree of each piece of evidence is measured. With Equation (4), the uncertainty degree measured by the IBE for the evidence is shown as follows:

$$\begin{array}{c} E_{ibe}\left(m_{0}\right)=-\sum_{SA1\subseteq \Omega }m_{0}\left(SAj\right)\log_{2}\left(\frac{m_{0}\left(SAj\right)}{2^{\left|SAj\right|}-1}e^{\frac{\left|SAj\right|-1}{\left|\Omega \right|}}\right)=2.0000\\ \begin{array}{c} E_{ibe}\left(m_{1}\right)=-\sum_{SA1\subseteq \Omega }m_{1}\left(SAj\right)\log_{2}\left(\frac{m_{1}\left(SAj\right)}{2^{\left|SAj\right|}-1}e^{\frac{\left|SAj\right|-1}{\left|\Omega \right|}}\right)=1.9710\\ E_{ibe}\left(m_{2}\right)=-\sum_{SA1\subseteq \Omega }m_{2}\left(SAj\right)\log_{2}\left(\frac{m_{2}\left(SAj\right)}{2^{\left|SAj\right|}-1}e^{\frac{\left|SAj\right|-1}{\left|\Omega \right|}}\right)=1.8464\\ E_{ibe}\left(m_{3}\right)=-\sum_{SA1\subseteq \Omega }m_{3}\left(SAj\right)\log_{2}\left(\frac{m_{3}\left(SAj\right)}{2^{\left|SAj\right|}-1}e^{\frac{\left|SAj\right|-1}{\left|\Omega \right|}}\right)=1.3568 \end{array} \end{array}$$

where j=1,2,3,4.

If evidence has a high degree of uncertainty, which is represented by a high value of IBE, then the reliability of the evidence is low, which means the evidence makes a small contribution to the final decision. Based on this cautious rule, the preprocessing of the evidence based on the uncertainty measure results will be based on the following function:

(7) $$m_{w}\left(SAj\right)=\sum_{i=0}^{3}m_{i}\left(SAj\right)\frac{e^{-E_{ibe}\left(m_{i}\right)}}{\sum_{i=0}^{3}e^{-E_{ibe}\left(m_{i}\right)}}$$

where j=1,2,3,4. With Equation (7), the preprocessing results at time T1, T2, and T3 are shown in Table 2.

Step 6: information fusion of air combat situation assessment and the prediction of military intention After evidence modification, information fusion of the situation assessment is based on Dempster’s rule of combination shown in Equation (3). Because the evidence modification is based on *n* (n=2,3,4) pieces of evidence at different times, the time of information fusion for the modified evidence in Table 2 is 1, 2, and 3 at time T1, T2, and T3, respectively. Take the SA1 as an example, the equations of evidence fusion results in Table 2 at time T1, T2, and T3 are shown as follows:

$$\begin{array}{c} m{\left(SA1\right)}_{\left(0,1\right)}={\left(m_{w}⊕m_{w}\right)}_{1}\left(SA1\right)=0.3002\\ m{\left(SA1\right)}_{\left(0,1,2\right)}={\left({\left(m_{w}⊕m_{w}\right)}_{1}⊕m_{w}\right)}_{2}\left(SA1\right)=0.4600\\ m{\left(SA1\right)}_{\left(0,1,2,3\right)}={\left({\left({\left(m_{w}⊕m_{w}\right)}_{1}⊕m_{w}\right)}_{2}⊕m_{w}\right)}_{3}\left(SA1\right)=0.9280. \end{array}$$

If the evidence in Table 1 is fused directly with Dempster’s rule of combination in Equation (3), the calculation equations for SA1 at time T1, T2, and T3 are as follows:

$$\begin{array}{c} m{\left(SA1\right)}_{T1}=\left(m_{0}⊕m_{1}\right)\left(SA1\right)=0.3002\\ m{\left(SA1\right)}_{T2}=\left(\left(m_{0}⊕m_{1}\right)⊕m_{2}\right)\left(SA1\right)=0.4600\\ m{\left(SA1\right)}_{T3}=\left(\left(\left(m_{0}⊕m_{1}\right)⊕m_{2}\right)⊕m_{3}\right)\left(SA1\right)=0.9280. \end{array}$$

In summary, the fusion results with evidence modification (shown in Table 2) and without evidence modification (shown in Table 1) are presented in Table 3.

**Figure 4:**
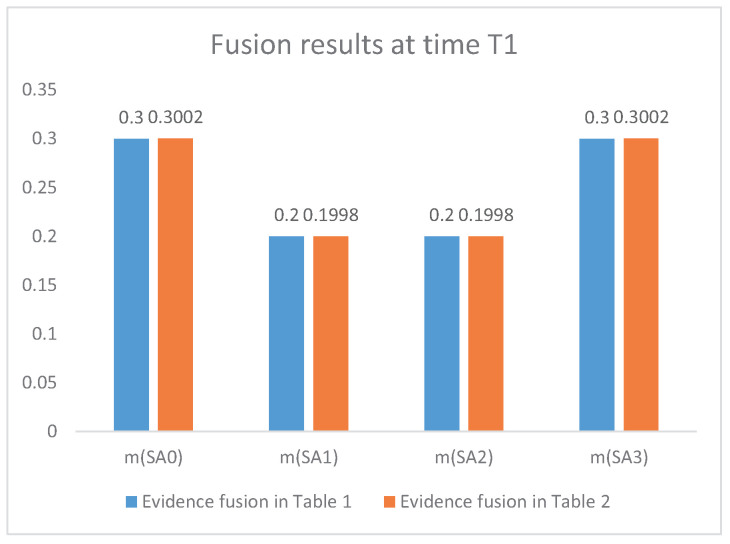
The fusion results of air combat situation assessment at time T1.

**Figure 5:**
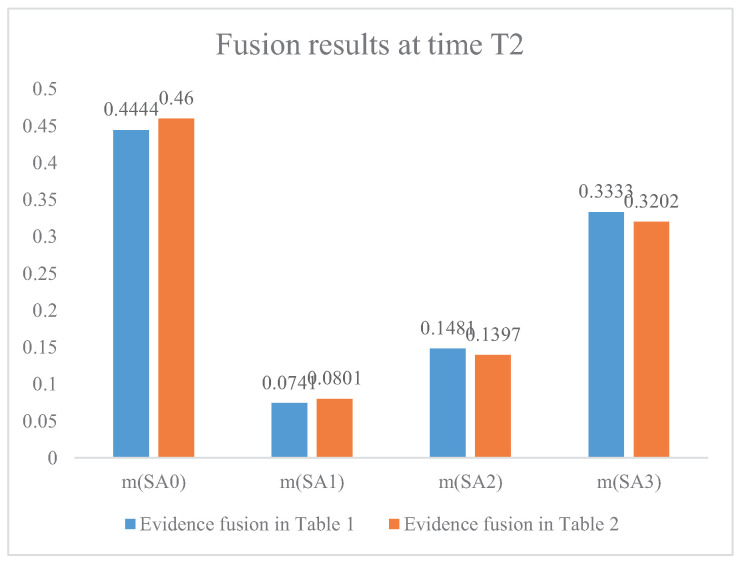
The fusion results of air combat situation assessment at time T2.

**Figure 6:**
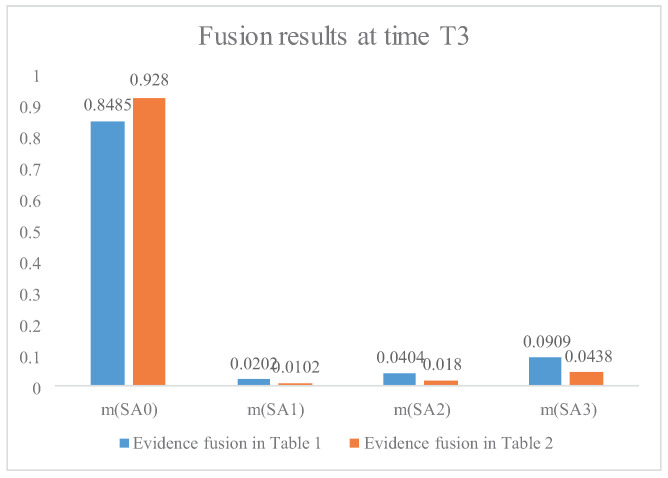
The fusion results of air combat situation assessment at time T3.

Figure 4, Figure 5 and Figure 6 present the comparison of the fusion results between the proposed method and the method without evidence modification, which means Dempster’s rule of combination is directly applied to the original evidence in Table 1. According to military knowledge, the fighter plane’s intention of attack becomes clearer from time T1 to time T3. The fusion result is consistent with judgements according to military knowledge. In addition, the fusion result based on the modified evidence in Figure 4, Figure 5 and Figure 6 has a higher belief on the situation SA1 at time T1, T2, and T3, respectively. Figure 6 shows that the proposed method has a belief of 92.80% with regard to the claim that the situation assessment result is SA1, which is nearly 8% higher than the fusion result without the IBE-based evidence modification. This is because the evidence modification procedure has a positive effect on the fusion result. The belief entropy measures the uncertainty of the situation assessment data and contributes to a positive effect on the fusion result-based decision-making for the pilots and commanders.

Step 7: real–time decision-making of air combat At time T3, with enough situation assessment information, the belief on SA1 is more than 90%, based on the proposed method. The decision support system will give the pilot a proposal on air combat situation SA1. However, if the evidence fusion is directly based on data in Table 1 without evidence modification, and the belief on air combat situation SA1 is only 84.85% at time T3, the decision support system cannot give a proposal because the threshold of the setting belief (90%) has not been reached. As for times T0, T1, and T2, the intelligent aided decision support system has not reached the belief of the threshold 90%; in this case, we recommend the belief on each candidate should be showed on the interface for reference of pilots.

## 5. Discussion and Open Issues

The IBE-based evidence modification can be regarded as an aided progress for the decision-making of pilots in air combat situation assessment. As is shown in Figure 6, the proposed method contributes to a higher belief in the potential air combat situation, which can be helpful for the decision-making of pilots. Although the uncertainty measure in the DST framework is applied to air combat situation assessment, some open issues need further study.

Firstly, the uncertainty in the battlefield environment, the acquired information from sensors and the air combat situation understanding according to the military knowledge of many experts can be transmitted to the process of evidence modeling. How the aforementioned uncertainty can be modeled in the DST framework to contribute to accurate evidence modeling needs a great deal of further study. In addition, apart from the IBE, there is potential for other solutions of uncertainty measure to solve this issue.

Secondly, the current example is artificial. How the initial beliefs are generated for the situation assessment information is an open issue related to the topic of generating mass function automatically. Currently, the existing method of generating mass function is based on specific applications [35,36]. In future research, algorithms such as the partially observable Markov decision process (POMDP) [37] and the hidden Markov model (HMM) [38,39] can be considered to determine or generate the initial mass functions.

Thirdly, the proposed method should be integrated to a simulation platform with air combat scenarios. The simulation platform should include each procedure in the framework of air combat situation assessment. Only in this way can the proposed method reach a state where it can be practically applied. In addition, in the simulation platform, the effectiveness of different methods can be compared visually and intuitively according to the final winning rate of a pilot.

Last but not least, complex application scenarios should be taken into consideration. In real applications, there may be more than two vehicles. How the current method can be extended to such complex situations needs further study.

## 6. Conclusions

To address the uncertainty of the situation assessment information in the Dempster–Shafer evidence theory framework, an IBE-based situation assessment method is proposed in this paper. Before applying evidence fusion, the uncertainty of the situation assessment in air combat is measured with the IBE, which contributes to a higher belief with regard to the final decision of the potential situation.

Apart from the open issues discussed in the paper, future work of this paper may focus on accurately measuring uncertainties in battlefield environments, evidence modeling situation sensing results in a DST framework, situation assessments involving open world assumptions, where there may be incomplete situations (the current work focuses on a complete FOD), and modeling and fusing conflict situation assessment information cautiously for reliable decision-making in air combat.

## Figures and Tables

**Table 1:** The mass functions of the situation assessment in air combat.

Evaluation Code	SA1	SA2	SA3	SA4
Situation Type	Attack	Defense	Escape	Feint
Time T0, $m_{0}$	0.25	0.25	0.25	0.25
Time T1, $m_{1}$	0.3	0.2	0.2	0.3
Time T2, $m_{2}$	0.4	0.1	0.2	0.3
Time T3, $m_{3}$	0.7	0.1	0.1	0.1

**Table 2:** Evidence modification of air combat situation assessment based on IBE.

Evidence Modification	$m_{0},m_{1}$	$m_{0},m_{1},m_{2}$	$m_{0},m_{1},m_{2},m_{3}$
m(SA1)	0.2754	0.3208	0.4623
m(SA2)	0.2246	0.1792	0.1496
m(SA3)	0.2246	0.2156	0.1725
m(SA4)	0.2754	0.2844	0.2156

**Table 3:** Fusion results of the air combat situation assessment based on the evidence in Table 1 and Table 2, respectively.

Evidence Fusion		$m_{0},m_{1}$	$m_{0},m_{1},m_{2}$	$m_{0},m_{1},m_{2},m_{3}$
Fusion of Table 1	m(SA1)	0.3000	0.4444	0.8485
	m(SA2)	0.2000	0.0741	0.0202
	m(SA3)	0.2000	0.1481	0.0404
	m(SA4)	0.3000	0.3333	0.0909
Fusion of Table 2	m(SA1)	0.3002	0.4600	0.9280
	m(SA2)	0.1998	0.0801	0.0102
	m(SA3)	0.1998	0.1397	0.0180
	m(SA4)	0.3002	0.3202	0.0438
